# The role of clinical trials units in maximising qualitative research with RCTS

**DOI:** 10.1186/1745-6215-14-S1-P99

**Published:** 2013-11-29

**Authors:** Cindy Cooper, Alicia O'Cathain, Danny Hind, Joy Adamson, Julia Lawton

**Affiliations:** 1 University of Sheffield, Sheffield, UK; 2 University of York, York, UK; 3 University of Edinburgh, Edinburgh, UK

## 

The value of qualitative research alongside RCTs is now widely accepted. Consolidated criteria for reporting qualitative research (COREQ) are well established (Tong et al. Int J Quality in Health Care 2007; 19 (6):349-357). A standard operating procedure for designing and implementing qualitative research with trials is available (Rapport F, *Trials* 2013, 14:54). However no guidance currently exists regarding staff related issues or reporting whilst the trial is in progress.

We reviewed the experience of two UKCRC registered CTUs in conducting qualitative research with trials and identified the following issues in need of debate:

1. Staff :-

a. recruitment – developing expertise ‘in-house’ versus contracting qualitative researchers with skills appropriate to individual trials

b. specialisation – emergence of the hybrid qualitative-trialist researcher

c. models of working - integration of staff within the trial management team versus autonomy and independence

d. perspectives - balancing contradicatory research paradigms (rigid/protocol driven vs flexible/open-ended)

2. The reporting of progress and outcome of the qualitative research whilst the trial is on-going:

a. feedback to the trial team: process; timing; purpose; who needs to know what

b. Measures to avoid unblinding

c. Dealing with issues of safety and participant dissatisfaction revealed in qualitative research without distorting fidelity of the intervention

d. Handling of interim findings which may necessitate changes to the qualitative research or main trial protocol.

e. Timing / nature of dissemination if the qualitative research completes much earlier than the whole trial.

We explore these issues and make recommendations for documentation within the trial protocol.

